# Predictors of Hip Fracture Among Patients With Atrial Fibrillation: A Retrospective Study

**DOI:** 10.7759/cureus.109449

**Published:** 2026-05-22

**Authors:** Izza Afzal, Mohammad Fahad Bin Imtiaz, Muhammad Sumeed Khalid, Muhammad Awais Bin Abdul Malik, Muhammad Ammar Ali, Sohaib Akbar, Mohammad Hamza Bin Abdul Malik

**Affiliations:** 1 Trauma and Orthopaedics, University Hospitals Dorset National Health Service (NHS) Foundation Trust, Poole, GBR; 2 Cardiology, Karachi Institute of Medical Sciences, Combined Military Hospital, Karachi, PAK; 3 Trauma and Orthopaedics, Benazir Bhutto Hospital, Rawalpindi, PAK; 4 Internal Medicine, Advent Health, Orlando, USA; 5 Internal Medicine, Northern Lincolnshire and Goole National Health Service (NHS) Trust, Scunthorpe, GBR; 6 Neurosciences, University Hospital Coventry and Warwickshire, Coventry, GBR; 7 Medicine, Rawalpindi Medical University, Rawalpindi, PAK; 8 Internal Medicine, Bahria University Medical and Dental College, Karachi, PAK; 9 Internal Medicine, Nassau University Medical Center, East Meadow, USA

**Keywords:** atrial, comorbidities, elderly, fibrillation, fracture, hip, predictor, retrospective

## Abstract

Background

Hip fractures represent a major public health concern among elderly individuals, with annual cases projected to exceed six million globally by 2050 due to population aging. Patients with atrial fibrillation (AF) may be at increased risk due to multiple comorbidities. Identifying those predictors of hip fracture in this population is essential for early risk stratification and prevention. Therefore, this study aimed to determine the predictors of hip fracture among patients with AF.

Methods

This retrospective study included all eligible 189 patients aged ≥50 years with confirmed atrial fibrillation, enrolled using consecutive sampling according to predefined inclusion and exclusion criteria. Demographic and clinical data were collected via a self-designed form. Patients were categorized into fracture and non-fracture groups. Statistical analysis was performed in the Statistical Package for the Social Sciences (SPSS) software, version 25 (Released 2017; IBM Corp., Armonk, New York) using independent t-tests and chi-square tests for group comparisons. Variables with clinical relevance were further analyzed using multivariate logistic regression to identify independent predictors.

Results

The mean age of participants was 67.60±8.26 years, and 27 (14.28%) patients developed hip fractures. Patients with fractures were significantly older and more likely to be female with lower BMI. Univariate analysis identified multiple significant associations (p<0.05). On further analysis, multivariate logistic regression confirmed age ≥65 years (odds ratio (OR)= 2.32, p=0.037), female gender (OR= 2.14, p=0.047), BMI <22 kg/m² (OR= 2.17, p=0.046), osteoporosis (OR= 4.37, p<0.001), vitamin D deficiency (OR=2.02, p=0.040), history of stroke (OR=2.24 , p=0.047), chronic kideny disease (CKD) (OR=2.12, p=0.048), polypharmacy (OR= 2.82, p=0.012), and prolonged anticoagulation (OR 3.14, p=0.007) as independent predictors. In contrast, smoking, physical inactivity, coronary artery disease, diabetes mellitus, and hypertension, lacked independent association with hip fracture within this population.

Conclusion

Hip fracture risk in patients with atrial fibrillation is independently associated with several demographic, clinical, and treatment-related factors. Advanced age, female gender, low BMI, osteoporosis, vitamin D deficiency, history of stroke, CKD, polypharmacy, and long-term anticoagulation are key independent predictors. Early identification and targeted management of these factors may help reduce fracture risk in this high-risk population.

## Introduction

Atrial fibrillation (AF) represents the most frequently observed persistent cardiac dysrhythmia in routine clinical settings and is linked to considerable worldwide morbidity and mortality [1].The occurrence of AF rises significantly with increasing age, rendering it especially prevalent among geriatric cohorts who frequently have multiple comorbid conditions. The incidence of AF demonstrates a pronounced age-dependent escalation, affecting <0.5% of individuals below 50 years of age and reaching 10% to 17% among persons aged 80 years or older [2].

Hip fractures constitute one of the most serious outcomes of falls among elderly individuals and are associated with marked disability, decreased autonomy, extended hospital admission, and heightened mortality [3]. Mortality rates following hip fracture have been reported up to 30% within the first year, while many survivors experience persistent mobility limitations, loss of independence, and long-term functional disability [4]. Worldwide, hip fractures are regarded as a key public health issue owing to their growing prevalence in aging cohorts and the considerable financial load on healthcare services. Post-fracture recovery is frequently partial, with many patients enduring lasting functional deficits and impaired quality of life. Annual hip fracture cases are projected to surpass six million globally by 2050, driven by population aging [5]. The vulnerability of older adults with cardiovascular disease is further highlighted by evidence showing that coronary artery disease (45.8%) and acute myocardial infarction (35.6%) are leading causes of sudden death in this population, underscoring their high-risk clinical profile. This elevated cardiovascular risk burden in patients with atrial fibrillation may also predispose them to additional adverse outcomes, including fragility fractures [1,6].

Patients with AF comprise an especially susceptible group for hip fractures owing to the convergence of several risk determinants. These include advanced age, frailty, polypharmacy, coexisting chronic illnesses, and anticoagulant therapy. Furthermore, AF is now widely acknowledged as an indicator of systemic pathology rather than a purely cardiac disorder [7,8]. Accumulating data indicate that AF correlates with persistent inflammation, endothelial impairment, diminished physical activity, and sarcopenia, each of which may undermine skeletal integrity and elevate fracture risk [9].

In addition to cardiovascular and systemic contributors, conventional osteoporosis-associated risk factors significantly influence fracture occurrence in AF patients. Diminished bone mineral density, vitamin D insufficiency, and low body mass index (BMI) are commonly documented in this geriatric cohort, and they additionally elevate fracture risk [10-12]. Moreover, concurrent conditions including diabetes mellitus, hypertension, chronic kidney disease (CKD), prior cerebrovascular events, and smoking contribute to generalized frailty and may indirectly heighten fall propensity and resulting hip fractures among individuals with AF [8,13-16]. Similarly, social determinants of health, including socioeconomic status, healthcare access, physical inactivity, and limited social support, may contribute to frailty, falls, and hip fracture risk in patients with atrial fibrillation, particularly in resource-limited settings [17].

Pharmacologic considerations are also particularly important in AF patients. Polypharmacy is common due to the need for multiple cardiovascular and non-cardiovascular medications, which increases the risk of drug interactions, dizziness, orthostatic hypotension, and impaired balance. These effects may contribute to falls and subsequent fractures [18]. AF exhibits a strong correlation with thromboembolic events, notably ischemic stroke, thus commonly necessitating prolonged anticoagulant treatment. Recent studies have also focused on improving stroke risk stratification in patients with AF, including those with cryptogenic stroke, through the development of novel risk prediction models and calculators [19]. Such approaches may support individualized anticoagulation strategies while balancing thromboembolic and bleeding-related risks. Although anticoagulation markedly decreases stroke incidence, it may concurrently increase hemorrhagic risk and possibly affect fall-associated sequelae, including fractures, particularly among frail geriatric individuals [20,21].

Despite the growing recognition of these determinants of hip fracture in patients with atrial fibrillation, limited evidence exists from developing countries regarding its predictors. Pakistan, with its rapidly aging population and rising burden of cardiovascular disease, is particularly vulnerable to the consequences of hip fractures. In light of the above, we hypothesized that demographic, clinical, comorbidity-related, and treatment-related factors may be associated with an increased risk of hip fracture in patients with atrial fibrillation. Therefore, the present study was conducted to identify the predictors of hip fracture in this population at a tertiary care hospital in Rawalpindi.

By identifying high-risk patients, healthcare providers can implement targeted interventions, such as bone mineral density screening, vitamin D supplementation, fall prevention strategies, multidisciplinary geriatric care, physical therapy, sarcopenia screening, nutritional optimization, and medication review/deprescribing strategies, to reduce the incidence of hip fractures and improve outcomes in this vulnerable population.

## Materials and methods

Study design and study population

This retrospective investigation was conducted at Benazir Bhutto Hospital, Rawalpindi, Pakistan, over a 24-month period from September 2023 to September 2025. A total of 189 patients with a confirmed diagnosis of AF were included using consecutive sampling according to predefined inclusion and exclusion criteria. All eligible patients during the study period were enrolled to ensure complete case capture and improve representativeness. Institutional ethical approval was obtained prior to the study’s initiation.

Inclusion and exclusion criteria 

The present study participants were included if they were aged 50 years or above, possessed a verified AF diagnosis, underwent routine clinical follow-up, had comprehensive medical documentation accessible for assessment, and received surveillance for hip fracture. Patients were excluded if they had a previous hip fracture, high-energy trauma, bone malignancy, or metabolic bone disorders other than osteoporosis (including Paget's disease, osteomalacia, and hyperparathyroidism).

Primary outcome and secondary outcomes 

The principal study outcome was the incidence of hip fracture among patients with atrial fibrillation. The secondary outcomes directed toward determining risk predictors of hip fracture among patients with AF.

Data collection 

AF was diagnosed according to established clinical guidelines, employing 12-lead electrocardiography (ECG) [9,15]. Hip fractures were confirmed via pelvic radiographs obtained in anteroposterior and standard lateral projections, and they were classified according to the Arbeitsgemeinschaft für Osteosynthesefragen/Orthopaedic Trauma Association (AO/OTA) classification system [5]. Osteoporosis was defined based on previously documented clinical diagnosis and/or dual-energy X-ray absorptiometry (DXA) findings (T-score ≤-2.5 in the hip or spine), as recorded in medical records [22]. Vitamin D deficiency was defined as serum 25-hydroxyvitamin D levels <20 ng/mL recorded in medical records [23]. Anticoagulants were not stratified in the analysis. Data acquisition encompassed demographic variables (age, sex), clinical profiles and comorbidities (BMI derived as weight in kilograms divided by height in meters squared, lifestyle factors, smoking, coronary artery disease, diabetes mellitus, hypertension, stroke, CKD), pharmacologic history (anticoagulant agents, polypharmacy defined as more than five medications), and bone health parameters (osteoporosis and vitamin D levels) obtained from institutional medical records using a self-designed data collection form. The form comprised two sections: the initial addressed history; the latter pertained to diagnostic investigation findings.

Data analysis

Statistical evaluation of data was performed with IBM SPSS Statistics, version 25 (2017; IBM Corp., Armonk, New York). Distribution normality for continuous parameters was evaluated via the Shapiro-Wilk test. Continuous variables were reported as mean±standard deviation, whereas categorical variables were summarized as frequencies and percentages. Comparative analyses employed the Chi-square test and the independent samples t-test. Univariate and multivariate logistic regression analyses were performed to identify independent predictors of hip fracture. Variables with clinical relevance and/or statistical significance in univariate analysis (p≤0.05) were included in the multivariate logistic regression model using the enter method. Statistical significance was defined as p<0.05.

## Results

The study included 189 patients with atrial fibrillation. The mean age was 67.60 years with a standard deviation (SD) of ±8.26 years, whereas mean BMI measured 24.22 with an SD of ±3.67. Of these participants, 108 (57.15%) were men and 81 (42.85%) were women. Overall, 27 patients (14.28%) sustained hip fractures. Seventeen (62.96%) patients experienced intertrochanteric fractures, while 10 (37.04%) patients suffered femoral neck fractures.

Table 1 summarizes the demographic and clinical profiles of the study cohort and compares parameters between participants with and without hip fractures. Results indicate that hip fractures occurred more commonly in elderly patients, notably those aged ≥65 years, alongside females and persons with reduced BMI. A markedly elevated prevalence of osteoporosis, vitamin D insufficiency, prior cerebrovascular accident, chronic kidney disease (CKD), polypharmacy, and prolonged anticoagulant therapy was observed in the hip fracture cohort (p<0.05). Although diabetes mellitus and hypertension exhibited greater frequency among hip fracture patients, these relationships did not achieve statistical significance (p>0.05). Likewise, factors including smoking history, physical inactivity, and coronary artery disease demonstrated no significant intergroup differences.

**Table 1:** Demographic and Clinical Characteristics of the Study Population With Comparison Between Hip Fracture and Non-Hip Fracture Groups p-values with the sign of (*) indicate significant variation of variables between study groups. Test statistic values with (t) is for independent t-test analysis while test statistic values with (χ² ) Chi-square test analysis.

Variable	Total (N=189)	Hip Fracture Group n=27 (14.28%)	Non-Hip Fracture Group n=162 (85.72%)	Test Statistic	p-value
Age (years) (Mean ± SD)	67.60±8.26	71.20±7.61	66.82±8.10	t=2.88	0.004*
Age Groups				χ²=4.17	0.042*
≥65 years	120 (63.49%)	20 (74.08%)	100 (61.72%)		
<65 years	69 (36.51%)	7 (25.92%)	62 (38.28%)		
Gender				χ²=2.45	0.045*
Male	108 (57.15%)	09 (33.34%)	99 (61.12%)		
Female	81 (42.85%)	18 (66.66%)	63 (38.88%)		
BMI (kg/m²) (Mean ± SD)	24.22 ± 3.67	22.30 ± 3.12	24.45 ± 3.50	t=2.19	0.034*
BMI <22 kg/m²	49 (25.92%)	16 (59.25%)	33 (20.37%)	χ²=5.62	0.018*
Smoking	52 (27.51%)	11 (40.74%)	41 (25.30%)	χ²=2.78	0.097
Sedentary Lifestyle	59 (31.21%)	12 (44.45%)	47 (29.02%)	χ²=3.10	0.078
Osteoporosis	63 (33.34%)	18 (66.67%)	45 (27.78%)	χ²=13.92	<0.001*
Vitamin D Deficiency	82 (43.39%)	17 (62.96%)	65 (40.12%)	χ²=4.86	0.029*
Coronary Artery Disease	70 (37.03%)	12 (44.45%)	58 (35.80%)	χ²=1.79	0.184
Diabetes Mellitus	76 (40.21%)	14 (51.85%)	62 (38.27%)	χ²=0.80	0.378
Hypertension	95 (50.26%)	15 (55.56%)	80 (49.38%)	χ²=0.39	0.550
History of Stroke	36 (19.04%)	14 (51.85%)	22 (13.58%)	χ²=4.05	0.042*
Chronic Kidney Disease (CKD)	34 (17.98%)	14 (51.85%)	20 (12.34%)	χ²=5.03	0.044*
Polypharmacy	77 (40.74%)	20 (74.07%)	57 (35.18%)	χ²=9.88	0.003*
Anticoagulation	71 (37.56%)	19 (70.37%)	52 (32.10%)	χ²=10.94	0.004*

Table 2 presents findings from univariate and multivariate logistic regression analyses evaluating hip fracture predictors. Univariate assessment revealed that age ≥65 years, female sex, low BMI, osteoporosis, vitamin D insufficiency, stroke, CKD, polypharmacy, and long-term anticoagulant therapy were significantly correlated with hip fracture. Following adjustment for potential confounding variables, multivariate assessment determined age ≥65 years, female sex, BMI <22 kg/m², osteoporosis, vitamin D insufficiency, polypharmacy (more than five medications), and prolonged anticoagulation as independent hip fracture determinants. Osteoporosis demonstrated the strongest independent association (odds ratio (OR) 4.37), followed by anticoagulation (OR 3.14) and polypharmacy (OR 2.82), while acknowledging that confidence intervals overlapped for the latter two variables. Stroke history and CKD also remained statistically significant post-adjustment. Conversely, smoking, physical inactivity, coronary artery disease, diabetes mellitus, and hypertension lacked independent association with hip fracture within this population.

**Table 2:** Univariate and Multivariate Logistic Regression Analysis of Predictors of Hip Fracture Among Patients With Atrial Fibrillation p-values with the sign of (*) indicate significant association between study variables. OR: Odds Ratio, CI: confidence interval

Variable	OR	95% CI	p-value	Adjusted OR	95% CI	p-value
Age ≥65 years	2.17	1.01-5.19	0.042*	2.32	1.20–5.08	0.037*
Female Gender	1.98	1.16-3.92	0.045*	2.14	1.32–4.16	0.047*
BMI <22 kg/m²	2.71	1.18-6.15	0.018*	2.17	1.04–4.66	0.046*
Smoking	2.05	0.91-4.64	0.097	1.67	0.76–3.65	0.203
Sedentary Lifestyle	2.79	0.92-5.20	0.078	1.79	0.84–3.78	0.130
Osteoporosis	4.32	1.92-9.74	<0.001*	4.37	1.94–9.95	<0.001*
Vitamin D Deficiency	2.55	1.09-5.96	0.029*	2.02	1.28–4.16	0.040*
Coronary Artery Disease	1.71	0.76-3.84	0.184	1.32	0.61–2.79	0.487
Diabetes Mellitus	1.47	0.68-3.22	0.378	1.26	0.60–2.62	0.569
Hypertension	1.29	0.58-2.87	0.550	1.19	0.56–2.46	0.663
History of Stroke	2.51	1.04-6.09	0.042*	2.24	1.20–4.12	0.047*
Chronic Kidney Disease (CKD)	2.62	1.12-5.56	0.044*	2.12	1.08–4.04	0.048*
Polypharmacy	5.32	2.25-12.76	0.003*	2.82	1.28–6.27	0.012*
Anticoagulation	4.25	1.77-10.06	0.004*	3.14	1.39–7.04	0.007*

## Discussion

This preset study evaluated the predictors of hip fracture among patients with AF and demonstrated that demographic, clinical, lifestyle, and treatment-related factors contribute to fracture risk in this population. The key findings indicate that advanced age, female gender, low BMI, osteoporosis, vitamin D deficiency, history of stroke, CKD, polypharmacy, and long-term anticoagulation are important contributors to hip fracture risk, with osteoporosis showing the strongest independent association. These findings highlight the multifactorial nature of fracture development in AF patients and support the concept of AF as a marker of systemic frailty rather than an isolated cardiac condition [8,13]. 

Age emerged as a significant predictor of hip fracture, with patients aged ≥65 years showing a higher risk. This finding is consistent with the established literature, as aging is strongly associated with decreased bone mineral density, sarcopenia, impaired balance, and increased fall risk. Studies from large population cohorts have similarly demonstrated that fracture risk increases significantly with age. In AF patients, this risk is further amplified due to overlapping frailty and comorbid disease burden [2,7]. 

The female gender also showed a significantly higher proportion of hip fractures. This trend aligns with global evidence indicating that postmenopausal women are at increased risk due to estrogen deficiency, which accelerates bone loss [10,14].

Low BMI was significantly associated with hip fractures in this study. Low body weight is a well-established risk factor for osteoporosis and fractures, as it reflects reduced mechanical loading on bone and lower protective soft tissue mass during falls. Similar findings have been reported in large cohort studies where underweight individuals had significantly higher fracture risk compared to those with normal or higher BMI. In AF patients, low BMI may also reflect underlying frailty and malnutrition, further contributing to poor bone health [10,13].

Osteoporosis demonstrated the strongest association with hip fracture in both univariate and multivariate analyses. This is consistent with the existing literature identifying osteoporosis as a major determinant of fragility fractures, though fall risk and other factors also contribute. Chronic inflammatory states, diminished physical activity, and hormonal changes contribute to bone resorption and reduced bone strength. In AF patients, systemic inflammation and limited mobility may further accelerate bone loss, explaining the strong observed association. These results of the present study are consistent with previous studies connecting AF with increased osteoporotic fractures [9-12].

Vitamin D deficiency was also significantly associated with hip fractures in patients with AF. Vitamin D plays a critical role in calcium metabolism, bone mineralization, and muscle function. Deficiency leads to impaired neuromuscular coordination and increased fall risk. Comparable relationships have been documented in prior investigations, wherein reduced vitamin D concentrations correlated with heightened falls and fractures in geriatric cohorts [5].

Comorbid conditions such as a history of stroke and CKD were significantly associated with hip fractures in patients with AF. Stroke increases fracture risk through gait instability, muscle weakness, and impaired balance, leading to a higher propensity for falls. This finding is backed by previous studies [15,16]. CKD contributes to chronic kidney disease-mineral and bone disorder (CKD-MBD), characterized by disturbances in calcium-phosphate metabolism, secondary hyperparathyroidism, and reduced vitamin D activation, ultimately resulting in decreased bone mineral density and poor bone quality. A similar role of CKD has been described in the literature in patients with AF [3,10, 24]. 

Polypharmacy also emerged as an independent predictor. The use of multiple medications increases the risk of drug interactions, sedation, orthostatic hypotension, and cognitive impairment, all of which predispose to falls. This is particularly relevant in AF patients who frequently require complex pharmacotherapy, and aligns with findings from prior studies in geriatric populations [1,18].

Anticoagulation was independently linked to hip fractures. Vitamin K antagonists such as warfarin may increase fracture risk directly by inhibiting osteocalcin γ-carboxylation and reducing bone strength. Indirect mechanisms include increased bleeding severity after falls and contributing to overall frailty. The risk profile of direct oral anticoagulants may differ, though this study did not distinguish between anticoagulant types. These findings align with studies from Taiwan and the United States emphasizing the importance of fall risk assessment and prevention in anticoagulated patients [20,21].

Although factors such as diabetes mellitus, hypertension, smoking, sedentary lifestyle, and coronary artery disease were not significantly associated with hip fractures in the present study, the existing literature suggests these variables influence fracture risk [1,8,12,15]. The lack of association in our cohort may reflect insufficient statistical power, confounding by stronger predictors, or population-specific differences.

These findings highlight the importance of comprehensive risk assessment in patients with AF. Routine evaluation should include bone health assessment, nutritional status, fall risk evaluation, and periodic medication review. Early identification of high-risk individuals may facilitate implementation of targeted preventive strategies, including vitamin D supplementation, osteoporosis management, physiotherapy, structured fall prevention programs, and optimization of polypharmacy. Particular attention should be given to elderly patients receiving long-term anticoagulation therapy.

This study has several limitations. First, its retrospective design limits the ability to establish causal relationships. Second, the single-center setting and consecutive sampling approach may limit external validity and introduce potential selection bias related to referral patterns and disease severity. Third, the relatively small sample size and limited number of fracture events may have affected the stability of the multivariable logistic regression model and increased the risk of overfitting. Fourth, residual confounding may exist due to unmeasured variables such as longitudinal bone mineral density changes, medication adherence, and detailed frailty indices. Therefore, further multicenter studies with larger sample sizes and prospective designs are warranted to validate these findings.

Despite these limitations, the study provides clinically relevant insights into the multifactorial nature of hip fractures in patients with AF within a South Asian population. The findings emphasize the importance of an integrated cardiometabolic and orthopedic care approach to reduce fracture risk and improve outcomes in this high-risk group.

## Conclusions

This study demonstrates that patients with AF who developed hip fractures were more likely to be older, women, and underweight, with a higher prevalence of osteoporosis and vitamin D deficiency. Osteoporosis showed the strongest association with hip fracture risk. In addition, comorbid conditions such as stroke and chronic kidney disease, along with treatment-related factors including polypharmacy and prolonged anticoagulation, were identified as independent associations with hip fracture in this population.

The key contribution of this study lies in defining an integrated, atrial fibrillation-specific hip fracture risk profile that combines demographic, clinical, comorbidity, and treatment-related factors, an area that remains insufficiently explored in existing literature. This provides a pragmatic framework for risk stratification in routine clinical practice.

Importantly, most of these predictors are identifiable through routine clinical assessment or basic laboratory testing, enabling cost-effective risk stratification. However, access to bone mineral density testing and vitamin D assays may be limited in some resource-constrained settings. Early identification and optimization of modifiable risk factors, including bone health evaluation and medication review, may help reduce hip fracture risk and improve outcomes in patients with atrial fibrillation.

## Appendices

**Table 3:** Research Proforma for Predictors of Hip Fracture among Patients with Atrial Fibrillation: A Two-Year Observational Study

Research Proforma for Predictors of Hip Fracture among Patients with Atrial Fibrillation: A Two-Year Observational Study		
Section-wise research questions	Options: write/tick the option	
Section A. (History-Based Variables)		
Demographic Details		
1. What was the age of the patient? (Years)		
2. What was the gender of the patient?	Male	Female
Clinical Profiles and Comorbidities		
3. What was the Body Mass Index (BMI) of the patient?	….. kg/m^2^	
4. What was the lifestyle of the patient?	Physically Active	Sedentary
5. History of smoking?	Yes	No
6. History of Coronary Artery Disease?	Yes	No
7. History of Diabetes Mellitus?	Yes	No
8. History of Hypertension?	Yes	No
9. History of Stroke?	Yes	No
10. History of Chronic Kidney Disease?	Yes	No
11. History of any chronic disease other than those mentioned above?	Yes	No
Pharmacologic History		
12. History of long-term Anticoagulation Therapy?	Yes	No
13. History of Polypharmacy? (defined as >5 medications),	Yes	No
Bone Health Parameters		
14. Presence of Osteoporosis?	Yes	No
15. Presence of Vitamin D deficiency?	Yes	No
Section B. (Investigations Reports-Based Variables)		
1. Presence of Atrial Fibrillation: confirmatory findings on Electrocardiogram?	Yes	No
2. Presence of Hip Fracture on X-ray?	Yes	No
3. What was the type of Hip Fracture?	Intertrochanteric Fracture	Femoral Neck Fracture
